# Dietary Polyphenols and In Vitro Intestinal Fructose Uptake and Transport: A Systematic Literature Review

**DOI:** 10.3390/ijms232214355

**Published:** 2022-11-18

**Authors:** Stefania Iametti, Francesco Bonomi, Mattia Di Nunzio

**Affiliations:** Department of Food, Environmental and Nutritional Sciences (DeFENS), University of Milan, Via Celoria 2, 20133 Milan, Italy

**Keywords:** polyphenols, fructose, uptake, transport, intestinal cells

## Abstract

Recent evidence links chronic consumption of large amounts of fructose (FRU) with several non-communicable disease. After ingestion, dietary FRU is absorbed into the intestinal tract by glucose transporter (GLUT) 5 and transported to the portal vein via GLUT2. GLUT2 is primarily localized on the basolateral membrane, but GLUT2 may be dislocated post-prandially from the basolateral membrane of intestinal cells to the apical one. Polyphenols (PP) are plant secondary metabolites that exert hypoglycemic properties by modulating intracellular insulin signaling pathways and by inhibiting intestinal enzymes and transporters. Post-prandially, PP may reach high concentrations in the gut lumen, making the inhibition of FRU absorption a prime target for exploring the effects of PP on FRU metabolism. Herein, we have systematically reviewed studies on the effect of PP and PP-rich products on FRU uptake and transport in intestinal cells. In spite of expectations, the very different experimental conditions in the various individual studies do not allow definitive conclusions to be drawn. Future investigations should rely on standardized conditions in order to obtain comparable results that allow a credible rating of polyphenols and polyphenol-rich products as inhibitors of fructose uptake.

## 1. Introduction

The use of high-fructose (FRU) corn syrup as a sweetener in beverages and processed foods (including sodas, fruit-flavored drinks, frozen desserts, and sports drinks) has increased by 33% worldwide during the last five decades [1,2]. FRU accounts for about 10% of total energy intake, or close to 50 g/day, in most affluent countries [3]. The World Health Organization recommends that FRU should not provide more than 10% of the total caloric intake but advises lowering this threshold to less than 5% [4].

Epidemiological studies have shown that excessive FRU intake induces adiposity and insulin resistance [5], which can lead to the development of type II diabetes mellitus (T2DM) [6] and non-alcoholic fatty liver disease [7]. In a recent cohort study, Yuan et al. reported that the excessive consumption of sugar-sweetened beverages and high total FRU intake was associated with an increased incidence and mortality of proximal colon cancer [8].

Dietary FRU is passively absorbed from the luminal side of the intestine by glucose transporter (GLUT) 5, one of the facilitative GLUT family members [9]. In addition, GLUT2, a low-affinity transporter that can also recognize glucose and galactose, may transport FRU at the apical epithelial intestine level, with a Km for FRU more than fivefold higher than that of GLUT5 [1]. After being taken up by intestinal epithelial cells, FRU is carried across the basolateral membrane by GLUT2 into the portal blood [10]. Unlike glucose, the liver almost entirely metabolizes the absorbed FRU by the sequential actions of fructokinase, aldolase B, and triose kinase. Although the liver metabolizes the majority of the ingested FRU, the intestine itself can metabolize up to 30% of an oral FRU load [11]. Due to the absence of feedback inhibition, almost all FRU absorbed is converted into hepatic triose-phosphate regardless of the cellular energy status [12]. Then, various pathways dispose of the resulting metabolites. Among these are oxidation, conversion into hepatic glycogen, or de novo lipogenesis [13]. Management and FRU absorption could provide a route for preventing post-prandial FRU-induced liver inundation and hence help to prevent some of the acute effects of FRU overload on liver metabolic processes.

A report dating to more than forty years ago indicated that the glycemic index in both normal and diabetic volunteers was inversely correlated with the polyphenol (PP) content of foods. Quite intriguingly, this effect of food PP does not relate to their antioxidant or redox properties [14]. Subsequent studies indicated that PP (and other phytochemicals) might show hypoglycemic properties by affecting different targets, including the modulation of intracellular insulin signaling pathways, increase in insulin secretion from β-cells, and the inhibition of intestinal enzymes and transporters [15,16]. Various clinical studies have shown that dietary PP consumption was able to prevent fructose-induced oxidative stress and insulin resistance in T2DM patients [17], as well as to attenuate hepatic insulin resistance in healthy men [18].

Post-prandially, the gut lumen has a concentration of PP roughly 10-fold higher than any other site in the body, making the gut lumen itself the first place where the effects of PP on sugar metabolism should be investigated and where an intervention to lower the threats associated with FRU overconsumption may be designed [19]. For example, one possible strategy to lower the risks stemming from the obesity/T2DM connection implies lowering the uptake of high-energy nutrients into enterocytes, thus acting before the energy overload caused by excessive FRU affects the body as a whole. In this framework, the inhibition of enzymes and transporters involved in the earliest steps of the FRU release/uptake sequence leads to increased concentrations of nutrients in distal sections of the small intestine, which—in turn—initiates the “ileal brake” [20]. 

Many studies have been carried out to investigate the effect of PP on glucose absorption in intestinal cells [21,22,23,24,25,26], as is also summarized in an authoritative review [27]. However, much less is known about the effects of PP on FRU absorption, and no comprehensive analysis of literature reports is available. Herein, we are trying to provide an overview of studies on the potential effect of PP and PP-rich products on FRU uptake and transport in cultured intestinal cells. Although human intervention studies remain the gold standard to evaluate the relationship between food components and health, the development of reliable in vitro/ex vivo models allow the investigation and identification of the cellular/molecular working mechanisms and represent a first—and undoubtedly necessary—step when investigating the health properties of food/food components.

This review also highlights the difficulties related to drawing coherent conclusions from studies carried out under highly variable experimental conditions and suggests ways of integrating the most appropriate methodologies and conditions into a possible unifying approach.

## 2. Methods

**Figure 1:**
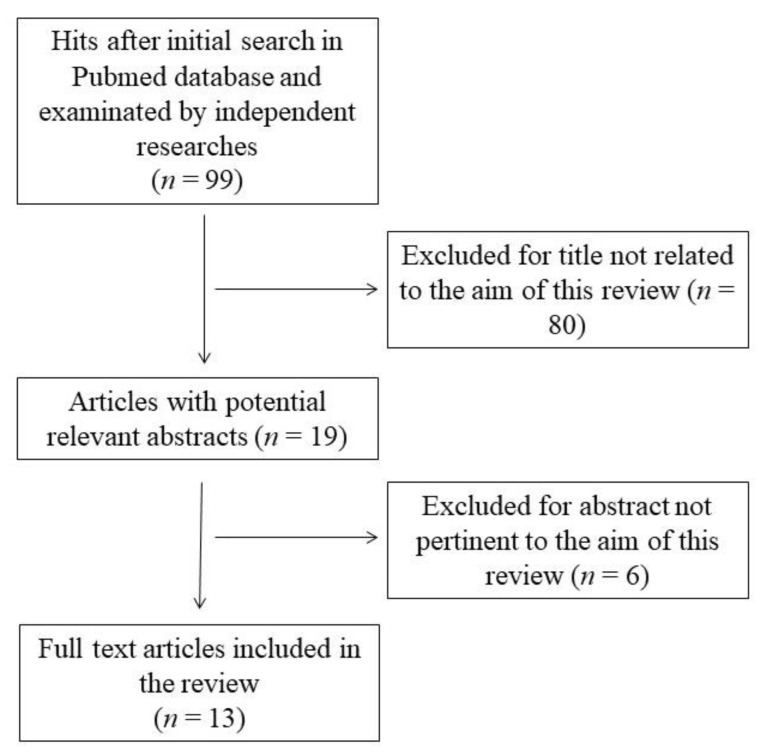
Flow chart of papers included in the review.

This systematic review was performed according to the Preferred Reporting Items for Systematic Reviews and Meta-Analyses guidelines (PRISMA) [28]. The search was carried out using the PubMed database in March 2022 and was conducted using the following keywords: “polyphenol” OR “phytochemical” AND “fructose transport” OR “fructose uptake” AND “intestinal cell” NOT “review”. The initial search yielded 99 hits. During the screening process (reviewing titles), 80 records were excluded. After abstract analysis, another 6 articles were excluded. Altogether, 13 records were selected and included in the review. Chosen studies were published between 2007 and 2022. Exclusion criteria were: (i) titles irrelevant to the research topic, (ii) abstract inappropriate or not related to the research topic, and (iii) experimental models other than enterocytes or intestinal tissues (i.e., *X. laevis* oocytes [29]). Reviews, letters, abstracts, and articles without a complete text in the English language were also excluded. Two independent investigators (S.I. and M.D.N.) checked the titles and abstracts of studies, and disagreements among the two reviewers were resolved through a mediator (F.B.). The detailed selection process is presented in Figure 1.

## 3. Results and Discussion

### 3.1. Effect of Single PP on FRU Uptake and Transport in Intestinal Cells

Table 1 summarizes the data published on the effect of individual PP species on FRU uptake (i.e., the amount of FRU absorbed by and measured in the cells) and on FRU transport (i.e., the amount of FRU absorbed by the intestinal cells in the apical chamber and released to the basolateral one).

The number of available studies is limited to nine cases. Six of them used Caco-2 cells [30,32,34,35,36,37]; two relied on Caco-2/TC7 cells [31,33], and only one was performed on an ex-vivo rat jejunum mounted in an Ussing chamber [38]. Most of these studies did not consider the simultaneous presence of more than one PP species (a common occurrence in a PP-rich diet) and the possible occurrence of competition/synergy among individual PP species [39].

The studies listed above cover a wide variety of PP. However, comparing the reported results is difficult because essential experimental conditions varied greatly between individual labs. The PP concentrations spanned a range from approximately 1 µM [35] to roughly 800 µM [31], whereas the FRU concentration covered an even broader span: from 100 nM [32,35] to 130 mM [31]. In addition, the methods and the duration of supplementation were not consistent. Some studies involved the pretreatment of intestinal cells with PP [32,33,35] in contrast with co-supplementation with FRU [30,31,34,36,37], whereas exposure times ranged from 5 min [37] to 24 h [35]. Despite these limitations, PP supplementation studies generally resulted in a concentration-dependent decrease in FRU uptake [32,34,35,36,37] and transport [30,31,33,34,38]. Also noteworthy is that supplementation with quercetin, apigenin, and chrisyn was reportedly associated with decreased expression of GLUT2 and GLUT5 mRNA [32,35].

### 3.2. Effect of Polyphenol-Rich Products on FRU Uptake and Transport in Intestinal Cells

Table 2 summarizes the data available on the effect of PP-rich products on FRU uptake and transport in intestinal cells.

Similar to studies on individual PPs, there are only a limited number of studies (9) evaluating the effect of extracts [30,32,33,36,40,41,42] and of a phenolic-rich juice [31,43]. All of these studies were conducted in cell lines (six with Caco-2 cells and three with Caco-2/TC7 cells), and the experimental conditions differed considerably among the various studies. The total PP concentration ranged from approximately 0.01 mg/mL [32,41] to 10 mg/mL [32], whereas the FRU concentration varied from 54 nM [41] to 130 mM [31]. Similarly, the method and duration of supplementation varied from co-supplementation with FRU for only 10 min [36] to pretreatment with phenolic extract for 24 h and subsequent incubation with FRU for 3 h [40]. Even though the use of different approaches makes it difficult to draw general conclusions, most studies reported a pronounced effect of most PP-rich products on both FRU uptake [32,36,40,41,42] and transport [30,33,42,43]. However, sugarcane extract supplementation was determined to result in an upregulation of GLUT5 mRNA and a downregulation of GLUT2 mRNA expression [32].

Given the substantial evidence for a role of FRU in the development of metabolic diseases and the many positive effects of the consumption of plant bioactives, there is an apparent need for assessing the effect of plant-derived food bioactives on FRU uptake. PP are compounds naturally synthesized by the secondary metabolism of plants and have piqued the interest of the scientific community because of their protective or preventive effects [44]. A partial list of the health-promoting effects of PP includes: anti-radical scavenging activity, the induction of antioxidant enzymes, anti-inflammatory action, anti-cancer properties, and gut microbiota regulation [45,46,47,48,49,50]. There is also extensive evidence regarding the influence of PP on intestinal sugar metabolism, with reported effects on various glycohydrolases as well as on sugar transporters, possibly leading to the modulation of their function and the alteration of glucose absorption [27]. In addition, PP may ameliorate inflammatory-induced intestinal permeability [51], thus decreasing transcellular sugar absorption [52].

FRU transport is mediated by members of the GLUT family of facilitated sugar transporters encoded by the solute carrier family 2A (SLC2A) genes, which are essential for intestinal FRU uptake [53]. Intestinal GLUT5 mRNA levels and FRU transport rates are shallow pre-natally and rapidly increase with weaning, independently of the diet. However, transporter levels can increase further following weaning compared to diets containing FRU [54]. FRU transport from the intestine to the blood is mediated by GLUT2 (SLC2A2), a high-capacity, glucose-dependent fructose co-transporter. GLUT2 is primarily localized on the basolateral membrane of enterocytes, but after feeding, GLUT2 may be dislocated from the intestinal basolateral membrane to the apical one by a vesicular mediated mechanism [55]. Considering the mechanisms underlying intestinal FRU uptake and transport, the inhibitory effects mediated by PP may result in a decrease of GLUT5 and GLUT2 expression levels, the inhibition of their activity, or suppressing the post-prandial translocation of GLUT2 from the basolateral membrane to the apical one.

### 3.3. Effect of Polyphenols on GLUT Family Expression

Although various studies have explored the effect of PP on GLUT2 expression [21,56,57], only a limited number of selected studies investigated the effect of PP and PP-rich compound supplementation on GLUT5 expression level [32,35,40]. High-FRU feeding induces intestinal thioredoxin-interacting protein (TXNIP), which binds and regulates GLUT5-mediated intestinal FRU transport [58,59]. Consistent with this, carbohydrate-responsive element-binding protein (ChREBP), a transcription factor that responds to intracellular carbohydrate nutrients and a known transcriptional regulator of TXNIP [60], also regulates intestinal GLUT5 and GLUT2 expression [61] and is required for systemic FRU tolerance [11,62]. Very recently, Zakłos-Szyda et al. found evidence that *Brassica juncea* leaf extract suppresses TXNIP, ChREBP, and GLUT5 mRNA expression in Caco-2 cells [40]. Based on these findings, it is reasonable to suspect that their downregulation may be implicated in the mechanism responsible for decreased FRU uptake by PP. Contrary to the general trend on the effect of PP and PP-rich products on intestinal FRU transporters, Ji et al. [32] reported enhanced GLUT5 mRNA expression after supplementation with sugarcane extract in Caco-2 cells. Since sugarcane contains several sugars, it cannot be excluded that the increase in GLUT5 mRNA expression may be due to other types of residual sugar in the extract that promote transporter expression [63]. However, Schreck et al. [41] found that most plant extracts did not affect FRU uptake. Possibly this might be due to the displacement of the radioactive substrates by non-radioactive monosaccharides during the absorption processes.

GLUT2 and GLUT5 expression are also regulated by intracellular signaling mechanisms depending on several kinase proteins, including protein kinase C (PKC), protein kinase A (PKA), phosphoinositide 3-kinases (PI3K), and p38 mitogen-activated protein kinases (MAPK) [64]. Although PP affect several distinct intracellular signaling pathways [65], Andrade et al. found evidence that the inhibitory effects of quercetin, apigenin, and chrysin upon FRU uptake and GLUT2 and GLUT5 mRNA expression level did not involve interference with any of these signaling pathways [35].

### 3.4. Polyphenols and GLUT Family Interactions

For the final effect on FRU uptake by PP, one should consider not only the expression of membrane transporters but also the direct interaction between PP and proteins involved in the release and uptake of simple sugars. In this framework, molecular docking simulation techniques may offer an easy way to evaluate the affinity of various ligands, including potential substrates and potential inhibitors, and are used for the theoretical prediction of the binding affinity and/or an analysis of the interaction sites [66]. Zakłos-Szyda et al. [40] found that procyanidin C1 had the highest inhibitory activity on FRU uptake despite having the lowest binding affinity with GLUT5 among the PP tested in this study. Although the lowest value of binding affinity indicates the highest stability of the resulting PP/protein complex, it does not unequivocally mean that the interactions will result in the inhibitory effect, due to the peculiar interactions involving receptors’ residues and inhibitors. An inspection of the amino acid residues and hydrogen bonding interactions suggests that the complex between procyanidin C1 and GLUT5 does not involve hydrogen bond interactions or affect a specific Ile residue in the binding site, which was previously indicated as one of the determinants of GLUT5 specificity to FRU [67].

### 3.5. Effect of Polyphenols on GLUT2 Translocation

To prove the role of PP in inhibiting GLUT2 translocation, Cohen et al. [68] performed time-lapse microscopy in GLUT2 transiently transfected MDCK II cells by addressing the glucose-induced internalization and the glucose-removal-induced externalization following 30 min of pre-incubation with 1 mM phloretin, a PP found in apple trees’ leaves [69]. These authors found that both the glucose-induced internalization and the glucose-removal-induced externalization of GLUT2 were inhibited by phloretin, thus suggesting the ability of the PP to block the translocation of the transporter to the apical membrane.

**Figure 2:**
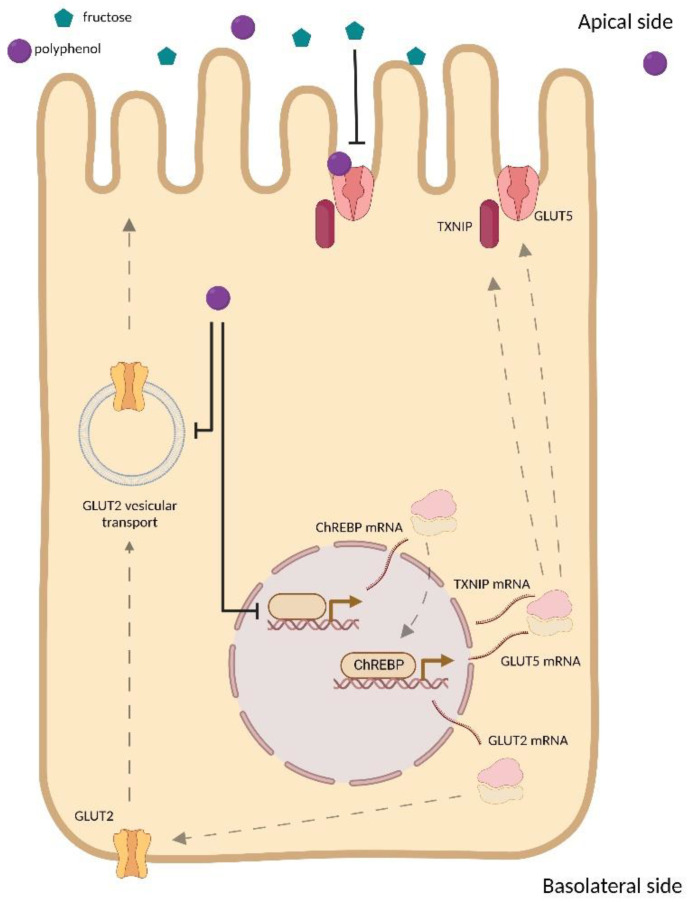
Summary of the proposed mechanisms for PP inhibition on FRU uptake.

A summary of the proposed mechanisms for PP inhibition on FRU uptake is illustrated in Figure 2.

## 4. Conclusions

In spite of the promising and encouraging effects on the intestinal absorption of FRU by PP and PP-rich compounds, the different experimental conditions adopted by the reviewed studies did not allow definitive conclusions to be drawn. Some essential considerations must be considered in future studies and are listed herein. (I) Several of the reports analyzed herein did not report the phenolic composition of the extracts used, making it impossible to attribute a possible inhibitory effect to particular categories of compounds. The inhibitory effect is not attributable only to a class of compounds but to many of them, different by group, chemical structure, and food source. (II) Most of the available studies have evaluated the inhibitory effect on FRU absorption with supplementation times from a few minutes [37] to 24 h [35,40] without considering that, at the intestinal level, the chyme can reside in the duodenum from 3 to 6 h [70]. In addition, several studies have used a PP concentration of a few or tens of μM [32,34,36,37], which is in sharp contrast with the concentration in the intestine after a PP-rich meal, which may reach the mM range [19]. The selection of a physiological conditions also appears particularly important in light of the well-evidenced concentration-dependent effect of PP [32,34,35,36,37,42,43]. (III) Further room for improving the current experimental approaches relates to the fact that most in vitro studies have evaluated glucose inhibition, considering only marginally the effect on fructose and almost disregarding those on sucrose [33,42]. Additional studies that evaluate the effect on these carbohydrates (not only at the level of absorption but—in the case of sucrose—also at the level of the saccharase enzyme) by PP are fundamental. (IV) Most studies use radiolabeled FRU without considering that, inside cells, this can be converted into metabolic intermediates containing radionuclides that can then be excreted at the basolateral level [71], thus leading to an overestimate of the transport of sugar. (V) Hesperidin mildly inhibited FRU transport in Caco-2/TC-7 cells, but the inhibitory effect of hesperidin was abolished in the presence of glucose and sucrose [31]. Future studies may wish consider the simultaneous presence of several sugars and the interplay among them. (VI) All of the studies reviewed herein have evaluated either individual phenolic molecules or whole food extracts without considering a possible matrix effect on PP bioaccessibility [72]. Future studies considering the food as is—and changes occurring during digestion—will be helpful to assess the efficacy of bioactive compounds present in food in inhibiting FRU uptake and transport in intestinal cells.

## Figures and Tables

**Table 1:** Summary of the information reported in the literature about the effect of phenolic compounds on intestinal FRU uptake and transport. FRU: fructose; NBDF: 1-deoxy-1-[(7-nitro-2,1,3-benzoxadiazol-4-yl)amino]-D-fructose; ↓: decrease; ↑: increase. Values between parentheses indicate the equivalent concentration expressed as molarity.

Ref.	Cell Model	Substrate and Concentration	Combination and Time of Incubation	Phenolic Compounds	Phenolic Concentration	Results
Sugimoto et al. [30]	Caco-2	1 mM FRU	Co-incubation for 3 h	Oenothein B	5 µg/mL (3.19 µM)	↓ FRU transport by 63%
				Gallic acid	50 µg/mL (293 µM)	↓ FRU transport by <20%
				Ellagic acid	50 µg/mL (165 µM)	↓ FRU transport by <30%
				Quercetin 3-O-b-D-glucoronide	50 µg/mL (104 µM)	↓ FRU transport by <30%
				Kaempferol-3-O-glucoronide	50 µg/mL (108 µM)	↓ FRU transport by <30%
Kerimi et al. [31]	Caco-2/TC7	130 mM ^14^C-FRU	Co-incubation for 30 min	Hesperidin	800 µM	↓ FRU transport approximately by 25%
Ji et al. [32]	Caco-2	100 nM NBDF	Pretreatment with compounds for 20 min and subsequent incubation with FRU for 30 min	Quercetin	0–100 µg/mL (0–331 µM)	↓ NBDF uptake in a concentration-dependent manner with a IC_50_ = 3.604 µg/mL (11.9 µM). ↓ GLUT2 mRNA expression in a concentration-dependent manner with a IC_50_ = 2.645 µg/mL (8.75 µM). ↓ GLUT5 mRNA expression in a concentration dependent manner with a IC_50_ = 1.788 µg/mL (5.91 µM)
Villa-Rodriguez et al. [33]	Caco-2/TC7	5 mM ^14^C-FRU	Pretreatment with compounds for 25 min and subsequent incubation with FRU for 60 min	Apigenin-7-O-glucoside	200 µM	↓ FRU transport approximately by 25%
				Apigenin	50 µM	↓ FRU transport approximately by 20%
				Apigenin-7-O-glucoside + apigenin	148 µM + 12 µM	↓ FRU transport approximately by 80%
				E-2-β-D-glucopyranosyloxy-4-methoxycinnamic acid, Z-2-β-D-glucopyranosyloxy-4-methoxycinnamic acid	Not reported	No effect
Satsu et al. [34]	Caco-2	200 nM ^3^H-FRU	Co-incubation for 10 min	Apigenin	10 µM	↓ FRU uptake approximately by 15%
				Kaempferol	10 µM	↓ FRU uptake approximately by 10%
				Tangeretin	10 µM and 25 µM	↓ FRU uptake approximately by 40% and 50% at 10 µM and 25 µM, respectively.
				Sinensetin	25 µM	↓ FRU uptake approximately by 55%
				Catechin gallate	25 µM	↓ FRU uptake approximately by 70%
				Nobiletin	10 µM, 25 µM, and 0–150 µM	↓ FRU uptake approximately by 40% and 65% at 10 µM and 25 µM, respectively. ↓ FRU uptake and transport in a concentration-dependent matter
				Epicatechin gallate	10 µM, 25 µM, and 0–150 µM	↓ FRU uptake approximately by 40% and 70% at 10 µM and 25 µM, respectively. ↓ FRU uptake and transport in a concentration-dependent matter
				Galangin, fisetin, myricetin, morin, puerarin, diosmin, flavonol, flavone, hesperetin, genistein, daidzein, naringin, naringenin, caffeine, catechin, epicatechin, epigallocatechin, epigallocatechin gallate, rutin, baicalein, flavanone, daidzin, glycitin, glycitein, quercitrin, quercetin, genistin, ginkgolides b, ginkgolides j, equol	10 µM	No effect
				Hesperetin, catechin, epicatechin, epigallocatechin, epigallocatechin gallate, gallocatechin, gallocatechin gallate	25 µM	No effect
Andrade et al. [35]	Caco-2	100 nM ^14^C-FRU	Pretreatment with compound for 20 min or 24 h and subsequent incubation with FRU for 6 min	Sinapinic acid	1 µM, 10 µM, and 100 µM	↓ FRU uptake approximately by 10% at 1 µM for 20 min.
				Ferulic acid	1 µM, 10 µM, and 100 µM	↓ FRU uptake approximately by 20% at 1 µM for 20 min. ↑ FRU uptake approximately by 15% at 1 µM and 10 µM for 24 h
				Caffeic acid	1 µM, 10 µM, and 100 µM	↓ FRU uptake approximately by 15% at 1 µM and 10 µM for 20 min. ↑ FRU uptake approximately by 15% at 100 µM for 24 h
				Coumaric acid	1 µM, 10 µM, and 100 µM	↓ FRU uptake approximately by 15% at 10 µM for 20 min.
				Proctocatenoic acid	1 µM, 10 µM, and 100 µM	↓ FRU uptake approximately by 15% at 10 µM for 20 min. ↑ FRU uptake approximately by 10% at 100 µM for 24 h
				Apigenin	1 µM, 10 µM, and 100 µM	↓ FRU uptake approximately by 20% and 25% at 10 µM and 100 µM for 20 min, respectively. ↓ GLUT2 mRNA and GLUT5 mRNA levels approximately by 90% and 75% at 100 µM for 24 h, respectively
				Chrysin	1 µM, 10 µM, and 100 µM	↓ FRU uptake approximately by 20% at all concentrations for 20 min. ↑ and ↓ FRU uptake approximately by 20% and 25% FRU at 1 µM and 100 µM for 24 h, respectively. ↓ GLUT2 mRNA and GLUT5 mRNA levels approximately by 90% and 75% at 100 µM for 24 h, respectively
				Hesperidin	1 µM, 10 µM, and 100 µM	↑ FRU uptake approximately by 15% and 20% at 1 µM and 10 µM for 24 h, respectively
				Naringenin	1 µM, 10 µM, and 100 µM	↑ FRU uptake approximately by 30% and 20% at 1 µM and 10 µM for 20 min, respectively. ↓ FRU uptake approximately by 15% at 100 µM for 24 h
				Rutin	1 µM, 10 µM, and 100 µM	↑ FRU uptake approximately by 10% at 1 µM for 20 min
				Quercetin	1 µM, 10 µM, and 100 µM	↑ and ↓ FRU uptake approximately by 20% and 25% at 1 µM and 100 µM for 24 h, respectively. ↓ GLUT2 mRNA and GLUT5 mRNA levels approximately by 90% and 75% at 100 µM for 24 h, respectively
				Kaempferol	1 µM, 10 µM, and 100 µM	↓ FRU uptake approximately by 15% at 100 µM for 24 h.
				Catechin	1 µM, 10 µM, and 100 µM	↓ FRU uptake approximately by 10% at 10 µM for 20 min.
				Epicatechin	1 µM, 10 µM, and 100 µM	↑ FRU uptake approximately by 25% at 10 µM for 20 min
				Epigallocatechin	1 µM, 10 µM, and 100 µM	↑ FRU uptake approximately by 25% at 10 µM for 20 min
				Delphinidin	1 µM, 10 µM, and 100 µM	↓ FRU uptake approximately by 15% at 1 µM and 10 µM for 20 min.
				Malvidin-3-glucoside	1 µM, 10 µM, and 100 µM	↓ FRU uptake approximately by 15% at 100 µM for 20 min, and at 10 µM and 100 µM for 24 h
				Cyanidin-3-glucoside	1 µM, 10 µM, and 100 µM	↓ FRU uptake approximately by 20% at 1 µM and 10 µM for 20 min.
				Gallic acid, ellagic acid, xantohumol, catechin, epigallocatechin gallate, delphinidin-3-glucoside, malvidin, resveratrol, sinapinic acid	1 µM, 10 µM, and 100 µM	No effect
Lee et al. [36]	Caco-2	10 mM ^14^C-FRU	Co-incubation for 10 min	Quercetin	0–30 µg/mL (0–99 µM)	↓ FRU uptake in a concentration-dependent manner with a IC_50_ = 29.64 µg/mL (98 µM)
				Catechin	0–30 µg/mL (0–103 µM)	↓ FRU uptake in a concentration-dependent manner with an IC_50_ = 87.08 µg/mL (300 µM)
				Curcumin	0–30 µg/mL (0–-81 µM)	↓ FRU uptake in a concentration-dependent manner with an IC_50_ = 65.57 µg/mL (178 µM)
				Bisdemethoxycurcumin	0–30 µg/mL (0–97 µM)	↓ FRU uptake in a concentration-dependent manner with a IC_50_ = 83.6 µg/mL (271 µM)
				Demethoxycurcumin	0–30 µg/mL (0–88 µM)	↓ FRU uptake in a concentration-dependent manner with IC_50_ = 37.43 µg/mL (110 µM)
Kwon et al. [37]	Caco-2	10 mM ^14^C-FRU	Co-incubation for 5 min	Quercetin	0–100 µM	↓ FRU uptake and transport in a concentration-dependent manner
		50 mM ^14^C-FRU	Co-incubation for various times (5–45 min)	Quercetin	200 µM	↓ FRU uptake and transport in a concentration-dependent manner
Andrade et al. [38]	Ex vivo rat jejunum in an Ussing chamber	1 µM ^14^C-FRU	Rats were fed for 18 weeks with the compound. Incubation of rat jejunum with FRU for 90 min	Chrysentin	100 mg/kg body weight	↓ FRU permeability approximately by 80%

**Table 2:** Summary of the literature information about the effect of PP rich products on intestinal FRU uptake and transport. FRU: fructose; GAE: gallic acid equivalent; NBDF: 1-deoxy-1-[(7-nitro-2,1,3-benzoxadiazol-4-yl)amino]-D-FRU; ↓: decrease; ↑: increase.

Ref.	Cell Model	Substrate and Concentration	Combination and Time of Incubation	Phenolic Compounds	PP-Rich Product	Phenolic/Extract Concentration	Results
Zakłos-Szyda et al. [40]	Caco-2	100 µM NBDF	Pretreatment with extract for 24 h and subsequent incubation with NBDF for 3 h	Gallic acid (3.96 mg/g), ferulic acid (7.25 mg/g), procyanidin C1 (70.43 mg/g), rutin (4.98 mg/g), quercetin-3-O-glucoside (0.45 mg/g), kaempferol-3-O-glucoside (10.4 mg/g), isorhamnetin-3-O-rutinoside (4.72 mg/g), isorhamnetin-3-O-glucoside (9.83 mg/g), kaempferol (30.97 mg/g), apigenin (165.81 mg/g)	Purified hydroalcoholic *Brassica juncea* (var. Green giant) leaf extract	0.25 mg/mL	↓ NBDF uptake approximately by 20%. ↓ GLUT5 mRNA and protein expression nearly by 20%
				Caffeic acid (2.02 mg/g), salicylic acid (24.63 mg/g), 3,5-dicaffeoylquinic acid (12.85 mg/g), ferulic acid (17.78 mg/g), sinapic acid (6.4 mg/g), procyanidin C1 (2.32 mg/g), epigallocatechin-3-gallate (51.62 mg/g), rutin (25.28 mg/g), quercetin-3-O-glucoside (27.70 mg/g), quercetin-3-O-glucuronide (22.2 mg/g), kaempferol-3-O-glucoside (18.56 mg/g), isorhamnetin-3-O-glucoside (1.05 mg/g), apigenin-7-O-glucoside (43.66 mg/g), apigenin (199.12 mg/g)	Purified hydroalcoholic *Brassica juncea* (var. Red giant) leaf extract	0.25 mg/mL	↓ NBDF uptake approximately by 30%. ↓ GLUT5 mRNA and protein expression nearly by 40% and 45%, respectively
				Chlorogenic acid (0.96 mg/g), caffeic acid (0.81 mg/g), 3-coumaric acid (0.17 mg/g), salicylic acid (28 mg/g), ferulic acid (36.2 mg/g), epigallocatechin-3-gallate (4.94 mg/g), quercetin-3-O-glucoside (1.9 mg/g), kaempferol-3-O-glucoside (6.21 mg/g), isorhamnetin-3-O-glucoside (20.17 mg/g), luteolin (6.45 mg/g), apigenin (40.47 mg/g)	Purified hydroalcoholic *Matricaria chamomilla* flower extract	0.25 mg/mL	↓ NBDF uptake approximately by 30%. ↓ GLUT5 mRNA and protein expression nearly by 25%, respectively
				Gallic acid (12.68 mg/g), 3-coumaric acid (6.24 mg/g), salicylic acid (41.88 mg/g), 3,5-dicaffeoylquinic acid (6.23 mg/g), ferulic acid (5.19 mg/g), procyanidin B2 (7.11 mg/g), (−)-epicatechin (6.27 mg/g), procyanidin C1 (256.76 mg/g), epigallocatechin-3-gallate (6.3 mg/g), epicatechin-3-gallate (6.51 mg/g), apigenin-7-O-glucoside (302.01 mg/g)	Purified hydroalcoholic *Apium graveolens* L., (var. Rapaceum) root extract	0.25 mg/mL	No effect
Sugimoto et al. [30]	Caco-2	1 mM FRU	Co-incubation for 3 h	Oenothein B, gallic acid, ellagic acid, quercetin 3-O-b-D-glucoronide, kaempferol-3-O-glucoronide	Hydroalcoholic *Eucalyptus globulus* leaf extract	1 mg/mL	↓ FRU transport by 65%
Schreck et al. [41]	Caco-2	54 nM ^14^C-FRU	Co-incubation for 1 h	Not reported	Methanolic *Juglans regia* leaf extract	1 mg/mL	↓ FRU uptake by 30.2%
					Methanolic *Peumus boldus* leaf extract	1 mg/mL	↓ FRU uptake by 32.6%
					Methanolic and aqueous *Adenophora triphylla* root, *Allium sativum* bulb, *Aronia melanocarpa* fruit, *Artemisia dracunculus* leaf, *Brassica oleracea* (var. Capitata alba) leaf, *Camellia sinensis* (var. Assam) leaf, *Camellia sinensis* (var. Darjeeling) leaf, *Camellia sinensis* (var. Gunpowder) leaf, *Camellia sinensis* (var. Sencha) leaf, *Ceratonia siliqua* fruit, *Citrus limon* fruit skin, *Coffea arabica* green seed, *Cornus officinalis* fruit, *Crataegus pinnatifida* fruit, *Cynara cardunculus* herb, *Eucommia ulmoides* bark, *Hibiscus sabdariffa* flower, *Ilex paraguariensis* leaf, *Lycium chinense* fruit, *Melissa officinalis* leaf, *Mentha aquatica* leaf, *Momordica charantia* fruit, *Nigella sativa* seed, *Olea europaea* leaf, *Origanum creticum* leaf, *Panax ginseng* root, *Potentilla aurea* herb, *Pueraria lobata* root, *Rosa rugosa* flower, *Rosmarinus officinalis* leaf, *Salvia officinalis* leaf, *Sarcopoterium spinosum* root, *Syzygium aromaticum* flower, *Thymus vulgaris* herb, *Vaccinium myrtillus* fruit, *Vitis vinifera* seed (pomace) extracts. Aqueous *Juglans regia* leaf, *Peumus boldus* leaf extracts	0.01–1 mg/mL	No effect
Ji et al. [32]	Caco-2	100 nM NBDF	Pretreatment with extract for 20 min and subsequent incubation with FRU for 30 min	Apigenin (1.89 µg/g), luteolin (5.3 µg/g), tricin (27.4 µg/g)	Hydroalcoholic sugarcane extract	0.01–10 mg/mL	↓ NBDF uptake in a concentration-dependent manner with a IC_50_ = 4.468 mg/mL. ↓ and ↑ of GLUT2 mRNA and GLUT5 mRNA expression in a concentration-dependent manner with a IC_50_ = 3.396 mg/mL and IC_50_ = 4.941 mg/mL, respectively
Villa-Rodriguez et al. [33]	Caco-2/TC7	5 mM ^14^C-FRU	Pretreatment with extract for 25 min incubation with FRU for 60 min	3-Caffeoylquinic acid (0.01%), 5-caffeoylquinic acid (0.07%), luteolin-7-O-glucoside (0.13%), umbelliferone (0.09%), di-caffeoylquinic acid (0.13%), apigenin-7-O-glucoside (12.3%), luteolin (0.01%) apigenin (0.28%), Z-2-β-D-glucopyranosyloxy-4-methoxycinnamic acid, E-2-β-D-glucopyranosyloxy-4-methoxycinnamic acid	Hydroalcoholic *Matricaria recutita* extract	1 mg/mL	↓ FRU transport by 28%
Villa-Rodriguez et al. [42]	Caco-2/TC7	5 mM ^14^C-FRU	Co-incubation for 60 min and pretreatment with extract for 25 min or 16 h and subsequent incubation with FRU for 60 min	3-Caffeoylquinic acid (0.01%), 5-caffeoylquinic acid (0.07%), luteolin-7-O-glucoside (0.13%), umbelliferone (0.09%), di-caffeoylquinic acid (0.13%), apigenin-7-O-glucoside (12.3%), luteolin (0.01%) apigenin (0.28%)	Hydroalcoholic *Matricaria recutita* extract	0–2 mg/mL and 1 mg/mL	↓ FRU uptake and transport in a concentration-dependent manner with a IC_50_ = 2 mg/mL and IC_50_ = 1 mg/mL, respectively. ↓ FRU uptake and transport approximately by 20% and 30% at 1 mg/mL for 25 min, respectively.
			Co-incubation for 60 min	(−)-epigallocatechin gallate (240 mg/g), (−)-epigallocatechin (70 mg/g), (−)-epicatechin (40 mg/g), (+)-catechin (17 mg/g).	Aqueous *Camellia sinensis* leaf extract	0–2 mg/mL	↓ FRU uptake and transport in a concentration dependent manner with a IC_50_ = 0.7 mg/mL and IC_50_ = 0.8 mg/mL, respectively.
Lee et al. [36]	Caco-2	10 mM ^14^C-FRU	Co-incubation for 10 min	Bisdemethoxycurcumin, demethoxycurcumin, curcumin	Acetonic turmeric extract	500 µg/mL	↓ FRU uptake approximately by 50%
				Catechin, quercetin	Aqueous guava leaf extract	500 µg/mL	↓ FRU uptake approximately by 50%
				Not reported	Hydroalcoholic rosemary extract	500 µg/mL	↓ FRU uptake approximately by 40%
				Not reported	Hydroalcoholic chrysanthemum, bayberry, Korea ginseng extracts. Aqueous onion, passionflower, touchi extracts	500 µg/mL	No effect
Kerimi et al. [31]	Caco-2/TC7	130 mM ^14^C-FRU	Co-supplementation for 30 min	Not reported	Orange juice	Regular strength	No effect
Moser et al. [43]	Caco-2	9 mM FRU + 3 mM d7-FRU	Co-incubation for 60 min	Gallic acid (5 mg/100 mL), caffeic acid (3.4 mg/100 mL), caftaric acid (4.2 mg/100 mL), quercetin-3,4-O-diglucoside (2.2 mg/100 mL), quercetin (16.8 mg/100 mL), isorhamnetin (4.9 mg/100 mL), piceid (0.8 mg/100 mL), resveratrol (7.1 mg/100 mL).	Grape juice (var. Niagara), harvested in 2013	10 µM, 50 µM, and 100 µM as GAE	↓ FRU transport by 10.5%, 28%, and 26.1% at 10 µM, 50 µM, and 100 µM, respectively.
				Gallic acid (1.9 mg/100 mL), caffeic acid (5.2 mg/100 mL), caftaric acid (9.6 mg/100 mL), epicatechin (1.5 mg/100 mL), quercetin-3,4-O-diglucoside (2 mg/100 mL), quercetin (19.8 mg/100 mL), isorhamnetin (6.2 mg/100 mL), piceid (5.3 mg/100 mL), resveratrol (13.3 mg/100 mL).	Grape juice with SO_2_ (var. Niagara), harvested in 2013	10 µM, 50 µM, and 100 µM as GAE	↓ FRU transport by 11.7%, 31.7%, and 29.5% at 10 µM, 50 µM, and 100 µM, respectively.
				Gallic acid (8.1 mg/100 mL), caffeic acid (11.1 mg/100 mL), caftaric acid (20.8 mg/100 mL), epicatechin (7.9 mg/100 mL), quercetin-3-O-glucoside (5.9 mg/100 mL), quercetin-3,4-O-diglucoside (4.2 mg/100 mL), quercetin-3-O-glucuronide (4.9 mg/100 mL), quercetin (30 mg/100 mL), isorhamnetin (12.6 mg/100 mL), piceid (2.2 mg/100 mL), resveratrol (3.5 mg/100 mL), cyanidin-3,5-O-diglucoside (623.5 ng/100 mL), cyanidin-3-O-glucoside (74.8 ng/100 mL), cyanidin-3-O-acetyl-glucoside (87.5 ng/100 mL), peonidin-3,5-O-diglucoside (144.7 ng/100 mL), peonidin-3-O -glucoside (13.6 ng/100 mL), peonidin-3-O-acetyl-glucoside (10.7 ng/100 mL), delphinidin-3-O-glucoside (620.5 ng/100 mL), delphinidin-3-O-acetyl-glucoside (113.2 ng/100 mL), delphinidin-3-O-p-coumaroyl-5-O-diglucoside (122.3 ng/100 mL), delphinidin-3-O-p-coumaroyl glucoside (199.8 ng/100 mL), petunidin-3-O-glucoside (191.1 ng/100 mL), petunidin-3-O-acetyl-glucoside (35 ng/100 mL), petunidin-3-O-p-coumaroyl-5-O-diglucoside (35.3 ng/100 mL), malvidin-3-O-glucoside (133.4 ng/100 mL), malvidin-3-O-acetyl-glucoside (22.3 ng/100 mL).	Grape juice (var. Concord), harvested in 2013	10 µM, 50 µM, and 100 µM as GAE	↓ FRU transport by 25.2%, 22.1%, and 30.9% at 10 µM, 50 µM, and 100 µM, respectively.
				Gallic acid (4 mg/100 mL), caffeic acid (4.2 mg/100 mL), caftaric acid (2.9 mg/100 mL), epicatechin (2.4 mg/100 mL), quercetin-3,4-O-diglucoside (2.5 mg/100 mL), quercetin (20.6 mg/100 mL), isorhamnetin (5 mg/100 mL), piceid (1.4 mg/100 mL), resveratrol (10.3 mg/100 mL).	Grape juice (var. Niagara), harvested in 2014	10 µM, 50 µM, and 100 µM as GAE	↓ FRU transport by 14.5%, 21.1%, and 41.3% at 10 µM, 50 µM, and 100 µM, respectively.
				Gallic acid (2.9 mg/100 mL), caffeic acid (7.6 mg/100 mL), caftaric acid (16.9 mg/100 mL), epicatechin (11.6 mg/100 mL), quercetin-3,4-O-diglucoside (3.3 mg/100 mL), quercetin (31.1 mg/100 mL), isorhamnetin (10.3 mg/100 mL), piceid (4.9 mg/100 mL), resveratrol (11.7 mg/100 mL).	Grape juice with SO_2_ (var. Niagara), harvested in 2014	10 µM, 50 µM, and 100 µM as GAE	↓ FRU transport by 12.9%, 23.3%, and 40.4% at 10 µM, 50 µM, and 100 µM, respectively.
				Gallic acid (8.9 mg/100 mL), caffeic acid (12.8 mg/100 mL), caftaric acid (25.1 mg/100 mL), epicatechin (12.6 mg/100 mL), quercetin-3-O-glucoside (7.8 mg/100 mL), quercetin-3,4-O-diglucoside (4.3 mg/100 mL), quercetin-3-O-glucuronide (3.8 mg/100 mL), quercetin (34.3 mg/100 mL), isorhamnetin (13.1 mg/100 mL), piceid (4.3 mg/100 mL), resveratrol (5.1 mg/100 mL), cyanidin-3,5-O-diglucoside (710 ng/100 mL), cyanidin-3-O -glucoside (106.9 ng/100 mL), cyanidin-3-O-acetyl-glucoside (40.8 ng/100 mL), peonidin-3,5-O-diglucoside (150.4 ng/100 mL), peonidin-3-O-glucoside (19.2 ng/100 mL), peonidin-3-O-acetyl glucoside (15.9 ng/100 mL), delphinidin-3-O-glucoside (877.4 ng/100 mL), delphinidin-3-O-acetyl-glucoside (210.3 ng/100 mL), delphinidin-3-O-p-coumaroyl-5-O-diglucoside (212.1 ng/100 mL), delphinidin-3-O-p-coumaroyl glucoside (211.7 ng/100 mL), petunidin-3-O-glucoside (210.1 ng/100 mL), petunidin-3-O-acetyl-glucoside (61.9 ng/100 mL), petunidin-3-O-p-coumaroyl-5-O-diglucoside (58.2 ng/100 mL), malvidin-3-O-glucoside (168.4 ng/100 mL), malvidin-3-O-acetyl-glucoside (38.8 ng/100 mL).	Grape juice (var. Concord), harvested in 2014	10 µM, 50 µM, and 100 µM as GAE	↓ FRU transport by 10.9%, 18.3%, and 18.1% at 10 µM, 50 µM, and 100 µM, respectively.

## Data Availability

Not applicable.
